# Bioelectrical Impedance Vector Analysis and Muscular Fitness in Healthy Men

**DOI:** 10.3390/nu8070407

**Published:** 2016-07-02

**Authors:** Fernando Rodríguez-Rodríguez, Carlos Cristi-Montero, Katherine González-Ruíz, Jorge Enrique Correa-Bautista, Robinson Ramírez-Vélez

**Affiliations:** 1 IRyS Group, Physical Education School, Pontifícia Universidad Católica de Valparaíso, Valparaíso 2374631, Chile; fernando.rodriguez@pucv.cl; 2 Physiotherapy program, Faculty of Health Sciences, Manuela Beltrán University, Bogotá 110231, Colombia; katherine.gonzalez@docentes.umb.edu.co; 3 Center of Studies in Physical Activity Measurements (CEMA Group), Faculty of Medical and Health Sciences, University of Bogotá, Bogotá 111221, Colombia; jorge.correa@urosario.edu.co

**Keywords:** bioelectrical impedance, phase angle, muscle fitness, handgrip strength

## Abstract

Muscle strength can define the general muscular fitness (MF) measurable through hand-grip strength (HG), which is a factor that relates to the health of people of different ages. In this study we evaluated the muscle strength together with a bioimpedance electric analysis in 223 healthy Colombian adult subjects. The bioelectrical impedance vector analysis (BIVA) was conducted to determine the resistance (R), reactance (Xc) and phase angle (PhA). We classified the subjects into three groups (for tertiles), obtaining lower values of R and Xc in subjects with lower HG, plus a high correlation between PhA and HG. An increase in the level of PhA is associated with a high level of MF in a sample of healthy Latin American adult men. The BIVA’s parameters and PhA are a potentially effective preventive measure to be integrated into routine screening in the clinical setting.

## 1. Introduction

The term muscular fitness (MF) has been used to represent muscular strength, local muscular endurance and muscular power [1]. Collective MF can be assessed using various strength performance tests such as handgrip (HG), explosive lower-limb power (jumps), and muscular endurance (sit-ups) [2]. Typically, HG strength can be measured using relatively inexpensive, portable and easy-to-use dynamometers [3]. In addition, low MF as determined with a HG dynamometer is recognized as a marker of poor metabolic profile during adolescence [4], and is associated with disease and mortality in adulthood [5]. Most current studies support an inverse relationship between MF and cardiovascular disease risk factors in youth, generally expressing muscular strength in relative terms [1,6].

Our group [1] and other researchers have shown that skeletal muscle strength is inversely associated with the incidence of coronary heart disease (CHD) [7], glucose intolerance and diabetes, [8] and in general, muscle strength is an indicator of general physical fitness, which is closely associated with the risk of CHD [9]. The HG strength provides useful information about overall muscular strength, and it could potentially be used in the clinical setting [1,7,8,9,10]. In general, HG strength correlates with the proportion of protein loss and muscle function, and, interestingly, it responds earlier to both nutritional deprivation and nutritional repletion than body composition parameters such as muscle or body mass [11].

Bioelectrical impedance analysis (BIA) has been widely used in hospitals and in research because it is a safe, non-invasive, convenient and economic technology [12]. BIA data (resistance (R); reactance (Xc)) through bioelectrical impedance vector analysis (BIVA) and phase angle (PhA) have been used to evaluate cellular function and hydration status [13], where R and Xc, separately considered, correlated highly with net fluid balance, without making any assumption on body composition [14]. Resistance is the decrease in voltage-reflecting conductivity through ionic solutions. The clinical application of BIVA methodologies has been highlighted due to the direct analysis of the bioelectrical properties of human tissue, which limits the potential error introduced by prediction equations used to estimate body composition [12,13]. Furthermore, cross-sectional data has shown age-related changes in whole-body BIVA variables, with increases in phase angle and decreases in Xc throughout the progression of adolescence [15], but decreases in both with advanced age [16] or more sexual maturation [17], and differences between individuals of varying training status/performance levels and sporting activities [18]. Norman et al. [13] reported that vector distribution patterns reveal the determinants of BIA results, i.e., the sex- and MF-dependency of BIA results indicate that equations used for the prediction of body composition from impedance measurements need to be validated separately by muscle function and MF classes.

Research associates MF with health, mainly in Caucasians [3,19], and there are also differences in strength in different population groups studied [20], influencing health status [21].

By comparing the HG strength between racial/ethnic groups (white, black and Hispanic), no significant differences can be seen when adjusting for age, height or body weight. However, when values are adjusted for lean mass, the white population shows greater strength [20]. These results indicate the importance of studying different populations, considering the differences in races [21].

The measurement of BIA in adults has been proposed to evaluate MF in men and women [12,13,14,15,16,17,18]. However, the application of BIVA to MF in Latin American adults has not been examined extensively [22,23,24]. Therefore, the purpose of this investigation was to examine the relationship between MF on BIVA in a sample of healthy Latin American men.

## 2. Materials and Methods

### 2.1. Subjects

A descriptive cross-sectional study was conducted in a population of healthy men. All subjects were from the Universidad of Rosario (Bogota, Colombia) and belong to different academic degree programs (masters and professional). We invited 223 men to participate in the study. The mean age of participants in the study was 27 years (±10). Participants were informed of the purpose of this study before they provided written consent to participate. The protocol was in accordance with the latest revision of the Declaration of Helsinki (as revised in Hong Kong in 1989 and in Edinburgh, Scotland, in 2000) and current Colombian laws governing clinical research on human subjects (Resolution 008430/1993 Ministry of Health), and was approved by the Institutional Review Board. After acceptance, a complete medical history, including family and personal history, was recorded. This study was conducted between January 2014 and December 2015. The following inclusion criteria were considered: age between 20 and 40 years; no movement restriction in the upper extremities; no self-reported history of inflammatory joint disease, neurological disorder or injury to the upper extremity; and not an athlete participating at an elite level. Subjects with a medical or clinical diagnosis of a major systemic disease (including malignant conditions such as cancer), type 1 or 2 diabetes mellitus, high blood pressure, hypothyroidism/hyperthyroidism, a history of drug or alcohol abuse, regularly using multivitamins, and inflammatory (trauma, contusions) or infectious conditions were also excluded from the study. The characteristics of the participants are shown in Table 1.

### 2.2. Anthropometric Assessment

Anthropometrics variables were measured by a Level 2 anthropometrist certified by the International Society for the Advancement of Kinanthropometry (ISAK), in accordance with the ISAK guidelines, in the morning following an overnight fast, at the same time (7 a.m.–10 a.m.). Body weight was measured in the subjects’ underwear and with no shoes, using electronic scales (SECA mBCA 515, Hans E. Rüth S.A, Hamburg, Germany) with a low technical error of measurement (TEM = 0.510%). Height (ht) was measured using a mechanical stadiometer platform (Seca 274, Hamburg, Germany; TEM = 0.019%). BMI was calculated as the body weight in kilograms divided by the square of height in meters. Waist circumference was measured at the midpoint between the last rib and the iliac crest using a tape measure (Ohaus^®^ 8004-MA, Parsippany, NJ, USA; TEM = 0.086%).

### 2.3. Handgrip Strength 

HG was measured using a standard adjustable handle Takei Digital Grip Strength Dynamometer Model T.K.K.540^®^ (Takei Scientific Instruments Co., Ltd., Niigata, Japan). Participants were given a brief demonstration and verbal instructions for the test, and, if necessary, the dynamometer was adjusted to the adult’s hand size according to predetermined protocols [1]. HG was measured with the subject in a standing position with the shoulder adducted and neutrally rotated and arms parallel but not in contact with the body. The participants were asked to squeeze the handle for a maximum of 3–5 s, and no verbal encouragement was given during the test. Two trials were allowed in each limb and the average score recorded the peak grip strength (kg). Thus, the HG values presented here combine the results of left- and right-handed subjects, without consideration for hand dominance. Since there is substantial covariance between strength capacity and body mass—and, moreover, the links between muscle strength and both physical function and chronic health are mediated by the proportion of strength relative to body mass—grip strength was normalized as strength per body mass (i.e., (grip strength in kg)/(body mass in kg)). All of the personnel were trained in testing and calibration procedures, and a calibration log was maintained. HG measurements in a subsample (*n* = 50, median age = 26.8 ± 2.4 years, 76.2 ± 12.4 kg, 1.65 ± 0.1 m, 22.9 ± 3.1 kg/m^2^) were recorded to ensure reproducibility on the day of the study. The reproducibility of our data was *R* = 0.96. Intra-rater reliability was assessed by determining the intraclass correlation coefficient (0.98, CI 95% 0.97 to 0.99). The study population was stratified HG value tertile groups (tertile 3, is the highest HG category) that could be displayed in the RXc graph.

### 2.4. Bioelectrical Impedance

Whole-body, tetrapolar, single-frequency (50 Hz) BIA (SECA mBCA 515, Hans E. Rüth S.A, Hamburg, Germany) was used to determine R (Ω), Xc (Ω), impedance (Z; Ω), and phase angle (°). The BIA device was calibrated with a 500 Ω test resistor (to verify R of 500 ± 5 Ω and Xc of 0 ± 5 Ω) per manufacturer guidelines. The reliability and validity of this system has been proved for Caucasian populations [18,19]. In the BIVA approach, introduced by Piccoli et al. [14] R (Ω) and Xc (Ω) normalized for ht, R/ht and Xc/ht, respectively, are plotted as a bivariate vector (RXc graph). In addition, measurements of R and Xc obtained at 50 kHz were normalized for the ht of the subjects (expressed in ohm per meter). In our study, two different BIVA approaches were used: the classic method, which involves standardizing the R and Xc values based on the subjects’ height [25], to remove the conductor length effect, and the new specific method, which involves standardizing the R and Xc values based on the cross-sections of the body together with the height, because of the assumption that the body impedance is affected by both the cross-sectional area and the height [26].

According to Piccoli et al. [14], R and Xc may therefore also reflect function in terms of, e.g., MF. Subjects stood on the metal contacts in bare feet, and body fat mass was determined. This measurement was repeated twice, and the average value was obtained. Hydration status with urine specific gravity values less than 1.025 was verified using handheld refractometry (Model CLX-1; VEE GEE Scientific, Inc., Kirkland, WA, USA).

### 2.5. Statistical Analysis

All data are given as mean and standard deviation. Multiple comparisons between the handgrip tertiles, anthropometric and BIVA values were compared using repeated measures analysis of variance with Bonferroni post-hoc tests. The correlation of changes in PhA and muscle strength was assessed through Pearson correlation. The mean impedance vector from classic and specific BIVA were compared with BIVA software using Hotelling’s T2 test to compare the muscular strength group between Xc//ht and R/ht and Mahalanobis distance (D) between groups defined by the two correlated variables and Hotelling’s (T2) test. An acceptable level of statistical significance was established a priori at *p* < 0.05. Statistical analysis was carried out using the software package Systat 21, SPSS Inc., Chicago, IL, USA.

## 3. Results

**Figure 1:**
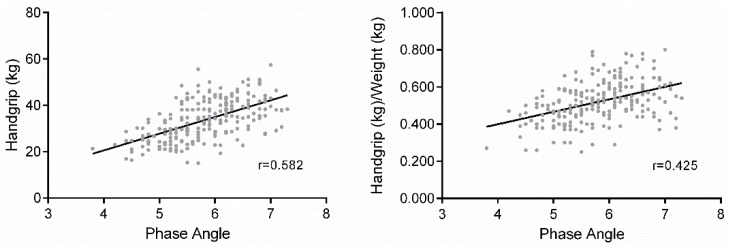
Partial correlation between muscle strength and phase angle in healthy men.

Table 1 shows descriptive statistics of anthropometric muscular strength and bioelectrical variables in the sample subdivided by MF tertile. A significant relationship is found between the BIVA parameters (R/ht and Xc/ht, *r* = 0.66), phase angle and HG (*r* = 0.582) and phase angle and HG/W (*r* = 0.425) of the study participants (Figure 1).

**Figure 2:**
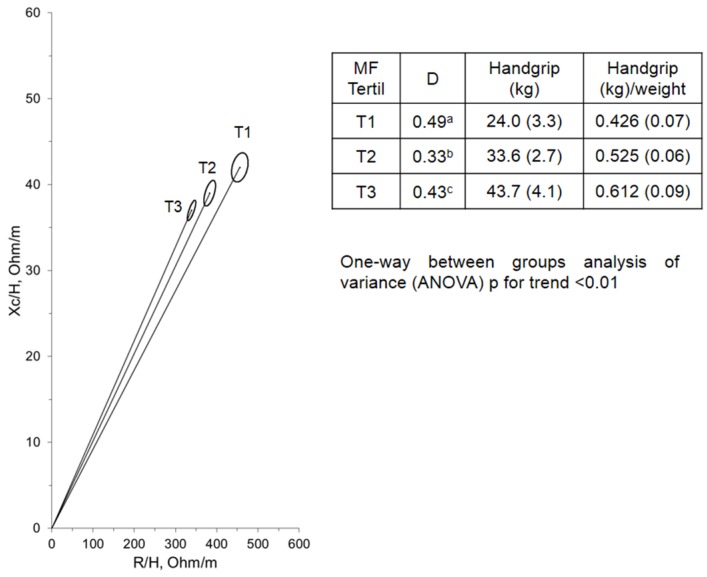
Significant average vector displacement of the handgrip strength, normalized as strength per body mass tertile groups. Individuals with different muscle strength (T1, lower tertile vs. T3, higher tertile) revealed significantly different bioelectrical characteristics. D = Mahalanobis distance between two groups defined by the two correlated variables. a = 1 vs. 2; b = 2 vs. 3; c =1 vs. 3. H, height. T2 = Hotelling’s statistic, 1 vs. 2, T2 = 9.6; 2 vs. 3, T2 = 6.1; 1 vs. 3, T2 = 2.2.

Figure 2 displays the mean impedance vectors of the HG which are normalized as strength per body mass tertile groups. A significant displacement of the vector due to both decreased Xc/ht and R/ht values with increasing HG was observed.

## 4. Discussion

The aim of this study was to examine the influence between HG on BIVA in a sample of healthy adult men. The main finding was a significant association between HG strength and the BIVA’s parameters, as well as PhA. Also, this study provides an important source of information both due to the lack of reference values in the Latin American population [22,23,24] and because most of the studies in this area have been made in geriatric, hemodialysis or HIV-infected patients [26,27,28].

Weak HG is also associated with high case fatality rates in individuals who develop any of a range of major illnesses, suggesting that low muscle strength may be an important indicator of vulnerability to disease and of frailty [2,3,4,5,6]. An analysis of body composition is an important screening tool for assessment and monitoring nutritional status. Traditionally, BIA has been use for the estimation of body composition, but nowadays its components (resistance and reactance normalized for height together with PhA) are being associated with several health conditions [12]. Indeed, the latter (PhA) has been considered a superior prognostic marker linked to an impaired nutritional and functional status, and is shown to be highly predictive of impaired clinical outcome and mortality in a variety of diseases and populations [27,28,29].

The results in the present study show that high PhA levels are associated with a better FM profile in healthy Latin American adult men. In particular, at the group (T3, higher muscular strength), shown high PhA levels (+12%, *d-cohen* = 1.37), than reference group (T1, low muscular strength). These results suggest that PhA is more closely correlated with lean body mass than with fat body mass in healthy adults. In fact, high PhA levels are an important marker related to cellular function and health, as well as muscle mass (due to the proportion of body water in this tissue) [12]. For example, men and healthy subjects have a higher level of PhA (around 5° to 7°), but sportsmen can reach much greater (9.5°) values [23]. The PhA mean in our sample was 5.8 ± 0.7° (27 ± 10 years old), 26.3% lower than that of a group of Mexican men (47.1 ± 16 years old; PhA 7.33 ± 0.88°), and 38.2% lower than that of a sample of Hispanic men (46.3 ± 18.3 years old; PhA 8.02 ± 0.75°) [20]. This result shows that even in a similar sample (race, age range), the values of PhA can be very different.

Skeletal muscle is one of the larger tissues of the body and is integrally involved in metabolic processes in both health and disease. However, muscle quality (a term that includes composition, metabolism, aerobic capacity, insulin resistance, fat infiltration, fibrosis and neural activation) appears to be more important than just muscle mass. This is because muscle mass alone cannot fully explain the loss of physical function [30], and muscle strength plays an important role in clinical practice [2]. For example, in the elderly, Rothenberg et al. [31] found that in spite of their low levels of muscle mass, they maintained normal values of HG strength and PhA [28]. High levels of strength are associated with muscle quality [29], a reduction in cardiometabolic risk [1], and mortality in men [5]. In turn, PhA has been positively associated with strength (HG and knee extension) [26], and skeletal muscle mass index (men: *r* = 0.52; women: *r* = 0.31) [32]. Hence, determining PhA could provide valuable information on muscle quality. In fact, a recent study in elderly women showed that a six-month resistance training program significantly increased PhA and decreased Rsp and R/ht. These outcomes had been associated with an improvement in body composition as well as a decrease in fat mass, an increase glycogen stores, and an improvement in cellular hydration [33,34,35].

On the other hand, most muscle mass in physically active men is mainly concentrated in the extremities [36]; therefore, both R and Xc values are lower in these muscle regions due to the amount of water and electrolytes, which promote electrical conductivity. Also, the values are greater in the region of the trunk. An important limitation of whole-body BIA is that the trunk contributes very little to R but contains a large content (~50%) of conductor volume. This could explain the underestimation of the percentage of body fat in adults with obesity by BIA [37]. In our study, we have shown that both impedance parameters Xc/ht and R/ht and thus vector migration are associated with changes of functional status as assessed by muscle strength using a handgrip dynamometer [10,11,12,13,14]. We interpret this as independent information about increased cell membrane surface and membrane integrity (Xc component) per unit of fluid volume (R component). The significant difference in XcSp (T-1: 32.8 vs. T-3: 55.2 cm/Ω, *p* < 0.05) in the current investigation and its relationship with strength suggests that this measure may be related indirectly to cellular adaptation and high muscle quality. XcSp has previously shown to be significantly related to skeletal muscle mass [10,12] and a predictor of both general health status and muscle strength [13]. In addition, the results of the current study showed that PhA and XcSp were significantly associated with HG group (T1, (low HG); *r* = 0.85, T2, (medium); *r* = 0.91, and T3 (high HG); *r* = 0.96). The quantitative distinction between contributions of PhA and XcSp by BIVA in T3 appears to be consistent with the existing literature in regards to muscle quality [36,38]. In this respect, vector displacements were likely the result of improved cellular integrity (XcSp) and cellular health (PhA).

Moreover, BIVA might be considered an interesting tool to reflect physiological function in the general population. This approach may be useful in monitoring the effects of nutritional support in subjects who cannot cooperate for grip strength measurements. Overall, because an increase in muscle mass has a greater influence in the extremities than the trunk [23], and because both lean and fat mass calculated from BIA using the published formula could differ substantially from the machine-generated values, it would be highly recommended to use correction factors to decrease the estimation error [36,37,38,39].

## 5. Conclusions

A decrease in R, Xc, R/ht, and Xc/ht values and an increase in the levels of PhA and XcSp are associated with a high level of MF in a sample of healthy Latin American adult men. The results of the current study strongly indicate that the vector position and migration is associated with function as assessed by HG (i.e., qualitative measures which reflect improved cell function and improved muscle function). BIVA’s parameters and PhA are a potentially effective preventive measure to be integrated into routine screening in the clinical setting. Further studies in the Latin American population (women, children, health condition), and using an analysis by segments with BIA, are warranted.

## Figures and Tables

**Table 1:** Anthropometric, muscle strength and bioelectrical impedance vector analysis (BIVA) characteristics of the study population by muscular strength tertile.

MF	*n*	ht	W	BMI	HG	HG/W	Total Body Water	Extra Cellular Water	Hydration	Phase Angle	R	Xc	R/ht	Xc/ht	R Sp	Xc Sp	*r*
		m	kg	Kg/m^2^	kg		L	L	%	°	Ω	Ω	cm^2^/Ω	cm^2^/Ω	cm/Ω	cm/Ω	
Tertile 1 (low HG)	79	1.60 (0.06) ^†^	57.5 (8.7) ^†^	22.3 (3.0)	24.0 (3.3) ^†^	0.426 (0.07) ^†^	32.2 (6.1) ^†^	13.5 (2.2)	73.3 (6.1) ^†^	5.2 (0.6) ^†^	732.9 (109.6)	68.8 (9.2) ^†^	456.7 (71.0) ^†^	42.9 (6.3) ^†^	488.0 (46.8) ^†^	32.8 (9.2) ^†^	0.33
Tertile 2 (medium HG)	71	1.69 (0.05) ^‡^	64.8 (9.7) ^‡^	22.6 (3.0)	33.6 (2.7) ^‡^	0.525 (0.06) ^‡^	35.1 (8.0) ^‡^	14.1 (2.9) ^‡^	68.4 (6.0) ^‡^	5.9 (0.6) ^‡^	647.6 (69.6)	66.8 (7.3) ^‡^	383.8 (47.2) ^‡^	39.5 (4.7) ^‡^	402.8 (51.7) ^‡^	45.5 (8.8) ^‡^	0.59
Tertile 3 (high HG)	73	1.72 (0.03) *	72.7 (10.2) *	23.6 (2.7) *	43.7 (4.1) *	0.612 (0.09) *	36.4 (7.3) *	15.3 (1.8) *	64.4 (64.2) *	6.2 (0.4) *	592.0 (54.9) *	64.7 (7.0) *	339.0 (35.3) *	37.1 (4.6) *	477.1 (55.7) *	55.2 (10.3) *	0.78
Total	223	1.68 (0.08)	65.0 (11.3)	22.8 (2.9)	33.8 (8.7)	0.52 (0.10)	36.4 (7.3)	14.6 (2.4)	67.3 (6.2)	5.8 (0.7)	659.6 (99.1)	66.8 (8.1)	395.0 (72.8)	39.9 (5.8)	428 (45.8)	45.3 (10.9)	0.66

Data given as mean (standard deviation). W: Weight; BMI: body mass index; HG: handgrip; HG/W: handgrip (kg)/Weight (kg); R: resistance; Xc: reactance; Sp: specific; ht: height; *r*: correlation coefficient between R/ht and Xc/h; R/ht resistance standardized for h; Xc/ht resistance standardized for ht. ANOVA showed significant difference between T1 vs. T2 ^†^; T2 vs. T3 ^‡^; T1 vs. T3 *, with Bonferroni post-hoc tests, *p* < 0.01.

## Abbreviations

The following abbreviations are used in this manuscript:

MF	Muscular fitness
HG	Handgrip
BIVA	Bioelectrical impedance vector analysis
PhA	Phase angle
R	Resistance
Xc	Reactance
